# Cost-Effectiveness of a Blended Learning Training Approach for Health Care Workers Conducting HIV Index Case Testing in Malawi: Secondary Economic Analysis of a Cluster-Randomized Controlled Trial

**DOI:** 10.2196/95450

**Published:** 2026-08-27

**Authors:** Poonam Rawat, Nora E Rosenberg, Katie R Mollan, Tapiwa A Tembo, Dhrutika Vansia, Jiayu Wang, Mike J Chitani, Maria H Kim, Ireen Chirombo, Caroline Kumbuyo, Victor Mwapasa, Katherine R Simon, Saeed Ahmed, Sarah E Rutstein

**Affiliations:** 1 Gillings School of Global Public Health University of North Carolina at Chapel Hill Chapel Hill, NC United States; 2 School of Medicine University of North Carolina at Chapel Hill Chapel Hill, NC United States; 3 Baylor College of Medicine Children's Foundation Lilongwe Malawi; 4 Department of Pediatrics Baylor College of Medicine Houston, TX United States; 5 Department of Epidemiology and Biostatistics Kamuzu University of Health Sciences Blantyre Malawi

**Keywords:** ACT, assisted contact tracing, blended learning, CEA, cost-effectiveness analysis, ICER, ICT, incremental cost-effectiveness ratio, index case testing, LMIC, low- and middle-income country

## Abstract

**Background:**

Index case testing (ICT) is an effective strategy for HIV case finding, but implementation in low- and middle-income countries (LMICs) is often limited by cost and logistical challenges. Traditional ICT training—centralized and in-person—is costly, disrupts service delivery, and varies in quality.

**Objective:**

This study evaluates the cost-effectiveness of a blended learning (BL) implementation package designed to build health care worker capacity for ICT, combining tablet-guided teaching and practice sessions, phone-based feedback, and tablet-guided continuous quality improvement, compared with standard of care (SOC) training.

**Methods:**

The Package of Resources for Assisted Contact Tracing: Implementation, Costs, and Effectiveness (PRACTICE) cluster-randomized controlled trial included 33 clusters in southern Malawi from May 2022 to September 2023, randomized 2:1 to SOC (n=22) or SOC + BL implementation package (n=11). Our cost-effectiveness analysis, from the health system perspective, used microcosting, time-and-motion assessments, and observed trial outcomes. The decision tree model estimated total program costs, contact testing outcomes, and incremental cost-effectiveness ratios (ICERs) per contact tested and per person diagnosed with HIV across 1 year of implementation. Sensitivity analyses assessed parameter uncertainty, and a scenario analysis modeled a nationwide scale-up under a decentralized, Ministry of Health–led approach.

**Results:**

Our model simulated 100,000 index clients eligible for contact elicitation over 1 year across 2 districts (50,000 per arm). The BL implementation package arm yielded 891 additional contacts tested and 54 more HIV diagnoses. The ICERs were US $125 per contact tested and US $2045 per person diagnosed with HIV; excluding training development costs reduced these to US $69 and US $1136, respectively. Nationwide scale-up under a Ministry of Health–led model further reduced ICERs to US $43 per contact tested and US $698 per person diagnosed with HIV. Probabilistic sensitivity analyses showed the BL implementation package was cost-effective in most simulations.

**Conclusions:**

The BL implementation package improved ICT delivery and HIV case finding. Scenario analyses suggested that a decentralized, government-led scale-up could substantially reduce costs and may represent an efficient case-finding strategy, particularly in Malawi’s mature epidemic. BL implementation packages provide a scalable, system-integrated, cost-effective approach that could strengthen health care worker capacity across other service delivery areas in low-resource settings.

**International Registered Report Identifier (IRRID):**

RR2-10.1136/bmjopen-2023-077706

## Introduction

Blended learning (BL), an educational approach combining in-person instruction with digital components, offers a promising and often more effective solution for strengthening health care worker (HCW) capacity in low- and middle-income countries (LMICs) [1-5]. It standardizes training content, enhances accessibility, and reduces the logistical burdens associated with traditional in-person training [3,6-11] and is supported by the World Health Organization (WHO) as a complement to face-to-face instruction [12,13].

One opportunity for BL is in the implementation of index case testing (ICT) for HIV case finding. Also known as voluntary assisted partner notification or contact tracing, ICT is an evidence-based intervention in which persons living with HIV, referred to as index clients, are supported in recruiting their contacts, including sexual partners and children, for HIV testing [14-17]. When implemented effectively, ICT plays a critical role in identifying persons living with HIV who need treatment services and HIV-uninfected individuals who may benefit from prevention services [18-21]. The WHO recommends ICT as a core component of comprehensive HIV testing and care [17]. Although Malawi and many other countries have adopted ICT into their guidelines [22,23], outcomes often fall short of those observed in clinical trials. In Malawi, limited HCW capacity and challenges with clinical coordination have hindered effective ICT implementation [15,18,22,24].

One key pillar for strengthening HCW capacity in LMICs is continuous training and quality improvement [25]. Historically, ICT capacity building has involved in-person training, role-playing, hands-on practice, and feedback. However, centralized, synchronous, face-to-face training poses significant challenges, including high costs related to travel, lodging, and per diems; disruptions to clinical services when multiple providers are away; limited opportunities for individualized practice or feedback; and variable quality due to differences in trainers’ teaching abilities [26-28]. Posttraining support, such as continuous quality improvement (CQI) activities, is often constrained by shortages of central staff, high travel costs, and difficulty monitoring progress across multiple facilities [14]. These barriers undermine the sustainability and consistency of ICT delivery.

To address these challenges, we developed a BL ICT implementation package that integrates digitally guided individual and small-group training to strengthen HCW capacity and improve ICT program fidelity [15,29]. The parent study, Package of Resources for Assisted Contact Tracing: Implementation, Costs, and Effectiveness (PRACTICE), assessed the feasibility, effectiveness, and cost-effectiveness of a BL implementation package layered on top of the standard of care (SOC) for Malawi’s ICT program, compared with SOC alone (hereafter referred to as SOC and SOC + BL, respectively). The study demonstrated improved fidelity to ICT protocols and more effective ICT outcomes [29]. Here, we examine the costs and cost-effectiveness of the BL implementation package for ICT implementation. We hypothesize that by improving HCW fidelity to ICT protocols and enhancing the effectiveness of ICT for reaching contacts of index clients, the approach may offer a cost-effective strategy for strengthening HIV prevention and care in Malawi.

## Methods

### Study Design and Setting

The PRACTICE study was developed using implementation science theory and formative qualitative research. Intervention design was informed by the Consolidated Framework for Implementation Research (CFIR) to identify barriers and facilitators to ICT delivery, while educational and behavioral theories and human-centered design principles guided the development and digitization of the BL implementation package. The resulting package was evaluated in a pragmatic cluster-randomized controlled trial (RCT) [15,16,30].

PRACTICE was a cluster-RCT conducted in the Machinga and Balaka districts of southern Malawi from May 2022 to September 2023. PRACTICE was described in detail previously [16]. Briefly, the study took place in 34 health care facilities, 14 in Balaka and 20 in Machinga, all of which routinely offer HIV testing and treatment services. These facilities included hospitals (n=3), health centers (n=27), and dispensaries (n=4), the latter being smaller facilities that provide basic primary health care. All facilities were operated by either the Malawi Ministry of Health (MOH) or the Christian Health Association of Malawi (CHAM), a faith-based organization. Both the MOH and CHAM provide HIV services free of charge under the national program. The selected sites received additional implementation support from the Tingathe Program, a US President’s Emergency Plan for AIDS Relief (PEPFAR)–supported initiative led by Baylor College of Medicine Children’s Foundation Malawi.

For randomization purposes, the 34 health care facilities were grouped into 33 clusters, with 2 neighboring facilities with similar baseline characteristics combined into a single cluster to allow for evenly sized allocation groups. Clusters were stratified by district, facility type, and the baseline number of potential index clients, then randomized in a 2:1 ratio to receive either SOC or SOC + BL.

### ICT Approach

ICT is an evidence-based approach used to identify individuals who may have been exposed to HIV. In Malawi, HCWs support persons living with HIV in identifying and testing their sexual partners and family members. This study considered index clients to be persons living with HIV who were either newly diagnosed through HIV testing services (HTS) at a facility or previously diagnosed and receiving antiretroviral therapy (ART). Potential contacts include the index clients’ sexual partners, biological children, household members, and social contacts [16].

At the time of the study, ICT in Malawi was implemented through three referral methods: (1) active or assisted referral, in which an HCW confidentially notifies and traces contacts for testing; (2) passive referral without self-test kits, in which the index client independently encourages contacts to test using a referral slip; and (3) passive referral with HIV self-test kits, in which the index client provides contacts with both a referral slip and an HIV self-test kit. Effective ICT implementation relies on trained lay HCWs to provide counseling and organize appropriate logistics for tracing contacts.

### SOC Implementation

The SOC consisted of 1 centralized, in-person, facilitator-led, 2-hour ICT training for HCWs, covering key ICT concepts, procedures, and counseling approaches. This one-time training was part of a larger Tingathe training that covered a range of areas beyond ICT. The SOC implementation was also supplemented by regular in-person facility mentorship visits conducted across all 33 Tingathe-supported clusters.

### BL Implementation Package for ICT

As previously described [16,29], clusters randomly assigned to the BL implementation package arm (n=11) received SOC + BL, which combined digital and in-person training with monthly CQI sessions. The intervention was structured into 4 phases. First, all HCWs completed an 8-hour asynchronous digital learning module over weeks 1 to 3 using tablets at their facilities. Following week 3, a 2-day (14-hour), in-person synchronous training was held over a weekend at or near their facility. These small-group, tablet-guided sessions included role-plays, individual feedback, and group discussions focused on ICT protocols. In weeks 4 to 5, all HCWs received a 1-hour phone feedback session based on simulated ICT encounters with study staff portraying index and contact clients; study staff provided feedback, and HCWs who struggled to master the material were given opportunities to repeat the exercises until their performance improved. Finally, from weeks 10 to 52, HCWs participated in a total of six 2-hour facility-based CQI sessions to review implementation, troubleshoot challenges, and refine strategies. In total, the BL components layered onto SOC accounted for approximately 35 additional hours of training, in addition to the 2 hours provided through SOC.

### Base Case

The base case represents the cluster-RCT scenario, modeling the estimated costs and outcomes for index clients enrolled in the 33 clusters participating in the PRACTICE study over 1 year [16,29]. The trial observed approximately 50,000 potential index clients receiving services at the 33 clusters per year. Potential index clients were defined as the sum of individuals enrolled in the ART program and those newly diagnosed through the HTS program over 1 year. To address the asymmetry of the 2:1 randomization, we simulated all 33 clusters receiving the intervention package (50,000 potential index clients) compared with all 33 clusters receiving the SOC (50,000 potential index clients), for a total of 100,000 index clients modeled.

Both study arms received standard ICT training and programming; thus, costs associated with the BL components were treated as incremental to SOC. SOC-related costs were assumed to be equivalent across arms and were not estimated separately.

Consistent with Malawi’s public health system, analyses were conducted from a health system perspective, incorporating system-level costs related to intervention implementation, tracing, and testing contacts of index cases living with HIV. We estimated the total incremental costs associated with developing and implementing BL over 1 year of operation. All costs and effectiveness outcomes were annualized to reflect this 1-year analytic time horizon. Our primary cost-effectiveness outcomes included cost per client tested and cost per person diagnosed with HIV, estimated using incremental cost-effectiveness ratios (ICERs).

### Probability Inputs

**Figure 1:**
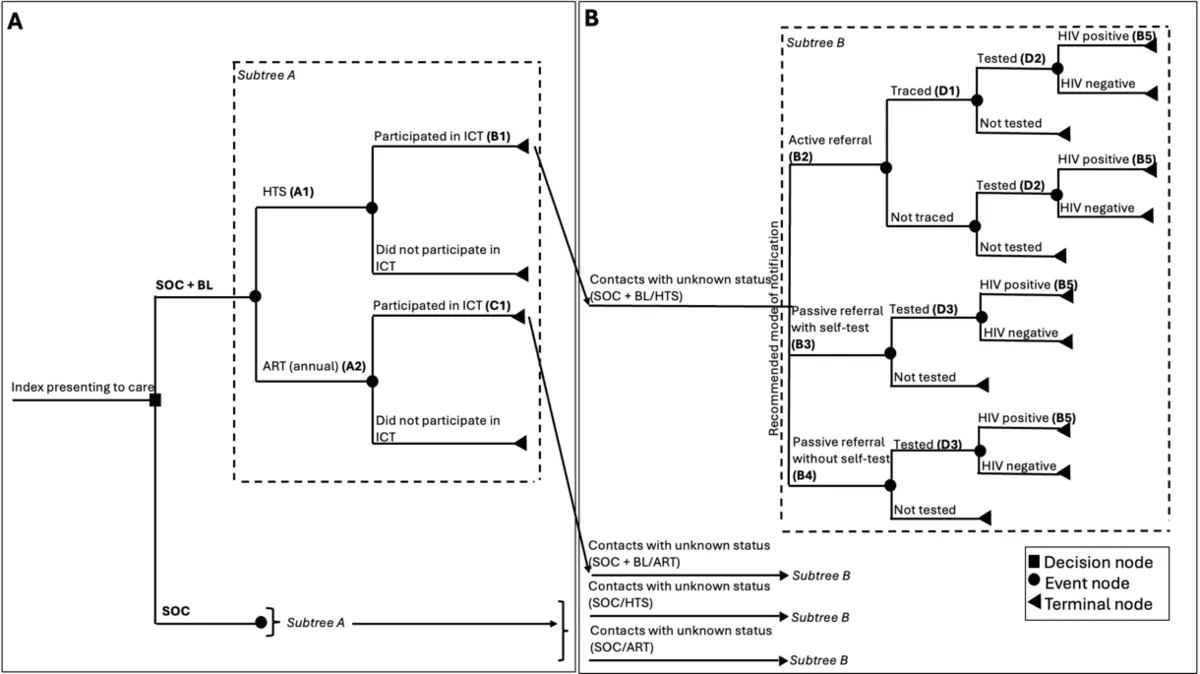
Decision analytic model for (A) index case testing pathways and (B) contact testing and HIV diagnosis following index elicitation. Standard of care (SOC) indicates standard index case testing (ICT) training and programming; SOC + blended learning (SOC + BL) indicates SOC supplemented with a BL implementation package. The letter labels (A, B, C, etc) on the nodes correspond to the probability values presented in Table 1. Each decision node represents a branching point where the sum of probabilities for all possible outcomes equals 1. For instance, the probability that an index client identified through HIV testing services (HTS) did not participate in ICT equals 1 minus the probability of participation (1 − B1). ART: antiretroviral therapy.

Event probabilities were estimated using observed clinical trial outcomes when available and supplemented by clinic register review and published literature. Plausible ranges were derived from reported 95% CIs, the minimum and maximum values found across relevant studies, or, when ranges were not explicitly reported, from the spread of values across comparable published estimates. When no published or empirical reference was available to inform parameter uncertainty, ranges were defined by applying a ±20% variation around the base-case value (Table 1 and Figure 1). The model was constructed to reflect the trajectory of index and contact client engagement with the health system and affiliated HCWs, from the point of index presentation to contact testing and HIV test outcomes.

**Table 1:** Model input parameters.

Parameter (decision node)^a^		Event probability value (plausible range), %		References
		SOC^b^	SOC + BL^c^	
**Index identification^d^**				
	Index identified via HTS^e^ (A1)	6.6 (6.6-18.2)	Same as SOC	Trial [31,32]
	Index identified via ART^f^ (A2)	93.3	Same as SOC	Trial, assumed
**Index case testing participation**				
	HTS group (B1)	92.9 (74.4-100)	97.1 (77.6-100)	Trial, assumed
	ART group (C1)	44.7 (36-54)	73.1 (58.4-87.6)	Trial, assumed
**Preferred contact elicitation method: HTS group**				
	Active referral (B2)	32.8 (31-100)	33.4 (26.4-39.6)	Trial [24], assumed
	Passive referral with self-test kits (B3)	7.4 (5.6-8.4)	7.5 (5.6-8.4)	Trial, assumed
	Passive referral (B4)	59.8 (30-100)	59.1 (47.2-70.8)	Trial, [24]
**Preferred contact elicitation method: ART group**				
	Active referral	37.7 (30-46)	47.7 (38.4-57.6)	Trial, assumed
	Passive referral with self-test kits	13.2 (10-16)	13.2 (10.5-15.8)	Trial, assumed
	Passive referral	49 (39-59)	39.1 (31.2-46.8)	Trial, assumed
**Tracing and testing^g^**				
	Tracing (D1)	100 (70-100)	Same as SOC	Assumed
	Testing: active referral (D2)	49 (40-62)	Same as SOC	Clinical record review [31]
	Testing: passive referral plus self-test kits (D3)	44 (16-71)	Same as SOC	Clinical record review [31,32]
	Testing: passive referral (D4)	15 (15-34)	Same as SOC	Clinical record review [31]
**HIV Positivity**				
	HTS group (B5)	9.6 (9.6-73.7)	9.1 (9.1-73.7)	Trial [31]
	ART group	2.9 (2.9-16)	4.2 (4.2-16)	Trial [31]

^a^The letter labels correspond to the specific decision nodes shown in Figure 1, connecting the probabilities in this table to their location within the decision tree.

^b^SOC: standard of care.

^c^SOC + BL: standard of care + blended learning.

^d^The probability of index client identification via ART is complementary to HTS (ie, 1 – HTS) and was assumed to be independent of intervention arm; therefore, overall trial proportions were applied to both study arms rather than arm-specific estimates.

^e^HTS: HIV testing services.

^f^ART: antiretroviral therapy.

^g^Probabilities assumed to apply across both HTS and ART groups.

### Cost Inputs

We included costs related to both developing and implementing the BL implementation package, including all 4 BL components described earlier (Multimedia Appendix 1). Costs were further designated as strategy development or implementation costs (Table 2).

**Table 2:** Cost inputs by activity and level (2023 US $).

Cost inputs			Level of cost incurrence		Total cost across 33 clusters (US $)
**Strategy development**					
	Content development	N/A^a^		22,000	
	Video production	N/A		10,719	
	Capital purchases^b^	District		14,951	
	Office supplies	Facility		1428	
	Miscellaneous^c^	N/A		386	
**Implementation**					
	Site-supervisor orientation (face-to-face)	District		3876	
	Individual tablet-guided learning sessions	HCW^d^		7839	
	Small-group face-to-face practice sessions	HCW or facility		31,950	
	One-on-one phone-based feedback	Facility		2013	
	Continuous quality improvement orientation (face-to-face and remote)	Facility		3394	
	Continuous quality improvement sessions and remote debrief (6 sessions)	Facility		11,927	
Total (fixed costs)			N/A		110,483
Tracing cost (per contact indicated for active referral)^e^			HCW		SOC + BL^f^: 0.90; SOC: 1.09

^a^Not applicable.

^b^Capital purchases include tablets and chargers.

^c^Miscellaneous includes data and time for preparing tablets for field use.

^d^HCW: health care worker.

^e^Tracing costs not included in fixed total. Applied dynamically in the model based on number of contacts traced in each arm.

^f^SOC + BL: standard of care + blended learning.

Cost data were obtained through microcosting and time-and-motion assessments. Microcosting relied on program receipts, program accounts, administrative records, and human resource records. Time-and-motion data were gathered through 3 distinct methods: self-reports and timed assessments from HCWs conducting field-based tracing (Multimedia Appendix 2), direct observations by study staff for phone tracing, and tablet-based paradata tracking of the time spent on digital learning tasks. Time estimates from each method were converted to costs using relevant HCW cadre salaries. In cases in which daily subsistence allowances were provided for a specific activity, we used the daily subsistence allowances as the cost input and did not double-count the associated salary. Costs were categorized at the district, facility, and HCW levels to inform scale-up projections (Multimedia Appendix 2).

Costs were calculated in 2023 Malawian Kwacha (MWK) and 2023 US $. The exchange rate used during the bulk of the trial period was US $1=MWK 1036 [33].

### Model Structure

We developed a decision tree model to simulate costs, contact tracing and contact testing outcomes, and estimate the ICERs comparing SOC with SOC + BL (Figure 1). Based on annual visit estimates for individuals presenting for HTS or ART care at 33 participating clusters, our base-case model reflects 50,000 potential index clients.

Our decision tree structure reflects the intentional separation between index and contact registers, in which index clients and contact clients are not directly linked to maintain confidentiality. As such, the model is presented in 2 subparts. Figure 1A reflects the movement of presenting index clients, beginning with a decision node (black square) assigning index clients to either the SOC or SOC + BL, using a 50:50 allocation. Within each arm, a chance node (black circle) determines whether the index client was identified via HTS or ART registers. This is followed by another chance node that models whether the individual participates in ICT, defined as naming contacts for tracing and testing.

Named contacts elicited from participating index clients then serve as the basis for the population moving through Figure 1B. The number of contacts elicited per index client was derived from trial data and varied by arm and source (SOC + BL/ART, SOC + BL/HTS, SOC/ART, and SOC/HTS). These estimates were applied externally by multiplying the number of index clients who participated in ICT (from Figure 1A) by the average number of contacts elicited per participating index client.

Once elicited, contact clients are categorized by HIV status as either unknown (requiring HIV testing) or known HIV-positive but not on ART. Contacts with unknown status proceed through 1 of 3 tracing methods according to the referring index client’s preference: active referral, passive referral with self-test kits, or passive referral without self-test kits. A series of chance nodes determine whether the contact is successfully traced, whether they undergo HIV testing, and, if tested, whether they receive a positive or negative result.

### Key Assumptions

We made several key assumptions that influence the model structure and potentially the outcomes. In this 2:1 cluster-RCT, only 11 clusters were exposed to the intervention. In our model, we present outcomes and associated costs as if all 33 clusters were exposed to the intervention, scaling district, facility, and HCW inputs to reflect this assumption. This is then compared with the SOC, again reflecting a scenario in which all 33 clusters received only standard services.

Further, we assumed that the proportion of index clients identified through either HTS or ART was independent of the intervention arm (Table 1). Finally, as the trial did not collect data on the proportion of named contacts who were traced, we used a base-case assumption that all named contacts in the active referral pathway were traced. The trial also did not collect testing rates by notification approach among named contacts; these estimates were obtained retrospectively through manual review of HIV testing registers at participating health facilities. All inputs were varied over a plausible range and tested in univariate and probabilistic sensitivity analyses to examine their relative importance, as described below.

### Analyses

We calculated ICERs by dividing the difference in costs by the difference in each outcome for the SOC + BL ICT implementation approach compared with the SOC approach.

We built the model in Microsoft Excel, version 16.97.2 (25052611), and used Crystal Ball (version 11.1.2.4.900; Oracle) to conduct probabilistic sensitivity analyses across 1000 simulations drawn from defined individual parameter distributions.

### Sensitivity and Scenario Analyses

To assess the robustness of the model, we conducted both 1-way (univariate) and probabilistic sensitivity analyses. The 1-way sensitivity analyses examined the impact of varying individual parameters on key outcomes to identify which inputs most influenced model results [34]. The probabilistic uncertainty analyses varied all parameters simultaneously based on their distributions, capturing the combined effect of parameter uncertainty on model outcomes [35]. We also conducted a sensitivity analysis of the US $–MWK exchange rate to assess the impact of fluctuations during the study period, which spanned October 2022 to September 2023.

We conducted 2 scenario analyses to explore the cost implications of alternative implementation approaches for the BL implementation package. First, we estimated implementation costs excluding one-time development costs, such as strategy design, content creation, and training materials, to simulate annual implementation across the original 33 clusters. This provided insights into the cost-effectiveness of a realistic implementation scenario in which development costs would not be duplicated, as these costs are otherwise included in the base-case approach.

Second, we modeled a nationwide rollout of the BL implementation package across all facilities providing HIV testing and ART services in Malawi over a 1-year implementation horizon. This scenario reflected a pragmatic, government-led implementation approach incorporating key efficiencies expected under MOH leadership by assuming integration within existing health system structures and personnel tasks. We used 2024 national data on new ART initiations and ongoing ART use to estimate the eligible index testing population, defining “initiations” as persons newly diagnosed with HIV (ie, HTS in our model). To estimate HCW needs, we scaled the number of HCWs per facility by client volume, using the base-case average (n=9) as a reference.

Implementation was envisioned through a decentralized cascade model. A 4-day central training of trainers (TOT) was modeled after existing programs for similar activities. The TOT was intended for district-level leadership, including 1 district health officer, ART coordinator, HTS coordinator, and driver from each district, along with 4 MOH staff members and 1 national trainer. Under this approach, efficiencies were expected through reduced reliance on external facilitators, use of existing MOH training spaces, and integration of ICT duties into the routine responsibilities of district and facility staff. Development costs were excluded, assuming use of already developed materials for national rollout. Cost categories were scaled based on facility, district, and personnel counts, with recurring costs (training delivery, travel, venues, and refreshments) adjusted to reflect district-led implementation. Fuel and venue costs were substantially reduced compared with the base case, consistent with local delivery and MOH-owned or subsidized training spaces. Detailed operational assumptions, including the composition of the TOT cohort, district-level training logistics, per diem and travel policies, and CQI delivery procedures, are provided in Multimedia Appendix 3.

To account for the possibility that training materials may require periodic updating or adaptation over time (eg, in response to evolving guidelines or contextual needs), we examined an additional scenario incorporating partial reuse of content development and video production materials (Multimedia Appendix 4). This scenario reflects reduced development costs associated with maintaining and updating existing materials rather than full redevelopment.

### Ethical Considerations

The PRACTICE trial was approved by the Malawi National Health Science Research Committee (#20/06/2566), the University of North Carolina at Chapel Hill Institutional Review Board (20-1810), and the Baylor College of Medicine Institutional Review Board (H-48800). All participating HCWs provided written informed consent prior to engaging in any study-related activities, including training sessions and program implementation.

## Results

### Overview

In the base case, we evaluated 50,000 potential index clients receiving services in the trial-included clinics over 1 year, modeling this cohort of 50,000 clients receiving SOC + BL compared with the same cohort receiving SOC. SOC + BL yielded a total of 9374 named contacts, compared with 7122 named contacts under SOC. Among these, we estimated that 3026 contacts would be tested and 183 individuals would be newly diagnosed with HIV under SOC + BL, compared with 2135 contacts tested and 129 individuals newly diagnosed under SOC. SOC + BL therefore resulted in 891 additional contacts tested and 54 more persons diagnosed with HIV (per 50,000) compared with SOC, representing approximately a 42% relative increase in both contact testing and HIV diagnoses. The total cost of implementing the BL strategy in the 33 clusters across 2 districts was US $111,258, resulting in an ICER of US $125 per additional contact tested and US $2045 per additional person diagnosed with HIV (Table 3).

**Table 3:** Cost-effectiveness outcomes under base-case and scenario assumptions.

Implementation strategy	Total cost of implementation (US $)	Eligible index clients over 1 year, n	Total contacts tested, n	Total persons diagnosed with HIV, n	Incremental contacts tested, n	Incremental persons diagnosed with HIV, n	ICER (US $ per contact tested)	ICER (US $ per person diagnosed with HIV
SOC^a^ (base case)	—	50,000	2135	129	—	—	—	—
SOC + BL^b^ (base case)	111,258	50,000	3026	183	891	54	125	2045
SOC + BL (excluding strategy development costs)	61,774	50,000	3026	183	891	54	69	1136
SOC (nationwide expansion)	—	957,654	40,897	2466	—	—	—	—
SOC + BL (nationwide expansion)	727,113	957,654	57,962	3508	17,065	1042	43	698

^a^SOC: standard of care.

^b^SOC + BL: standard of care + blended learning.

### Sensitivity Analyses

One-way sensitivity analyses identified the key drivers of uncertainty in the cost-effectiveness model. For cost per contact tested, ICERs were most sensitive to probabilities within the SOC condition, specifically, the probability that index clients identified through ART services would engage in ICT and the probability that index clients identified through HTS (ie, newly diagnosed) would opt for passive referral for their named contacts. Variations in these parameters shifted the estimated ICER for contact testing by up to ±US $34, indicating substantial leverage on cost-effectiveness.

For cost per person diagnosed with HIV, ICERs were again primarily driven by probabilities estimated in the SOC condition, specifically, the probability that an index client identified through HTS would choose passive referral and the probability of ICT enrollment among individuals identified through HTS. These parameters produced the largest ICER swings for this outcome, shifting the estimated cost per person diagnosed with HIV by ±US $850. Together, these findings indicate that both cost per contact tested and cost per person diagnosed with HIV are far more sensitive to programmatic outcomes than to associated implementation costs. Overall, these findings highlight that the robustness of the cost-effectiveness estimates depends on accurate empirical data for referral pathways and ICT uptake.

The full table of 1-way sensitivity analysis results comparing SOC and SOC + BL, along with tornado diagrams showing percentage changes in ICERs across plausible parameter ranges, are provided in Multimedia Appendices 5 and 6.

**Figure 2:**
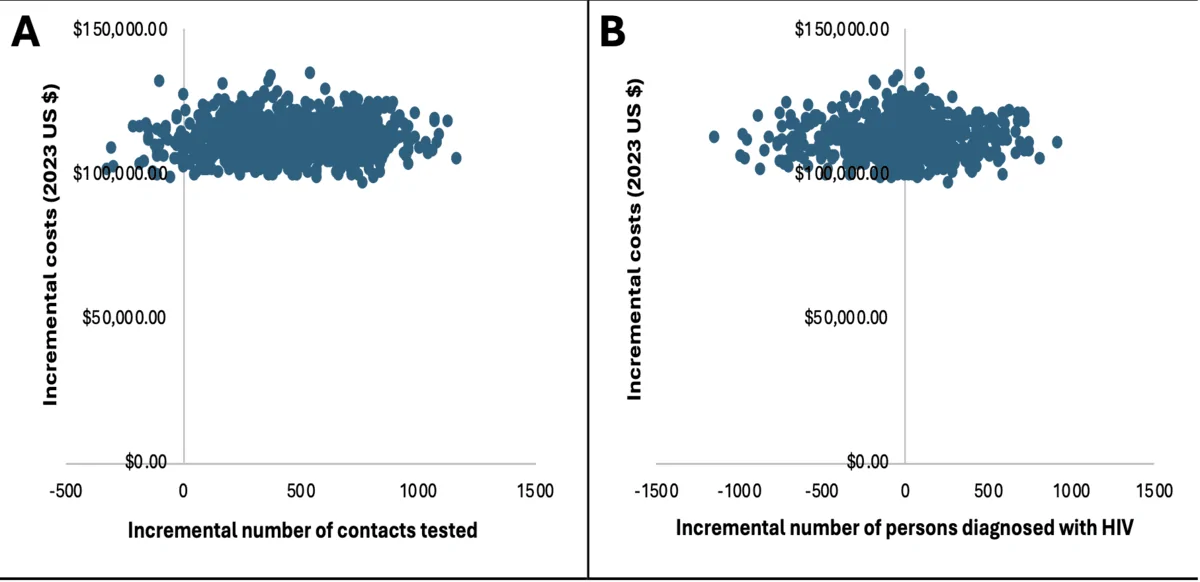
Incremental cost-effectiveness ratio (ICER) plane (base-case) showing (A) incremental cost vs contact tested and (B) incremental cost vs persons diagnosed with HIV. The 4 quadrants on the ICER plane indicate different cost-effectiveness outcomes: northeast (more costly, more effective), northwest (more costly, less effective), southeast (less costly, more effective), and southwest (less costly, less effective). This figure presents the base-case scenario summarized in Table 3.

Figure 2 presents cost-effectiveness planes from the probabilistic sensitivity analysis, showing the joint distribution of incremental costs and effects for SOC compared with SOC + BL. Figure 2A plots incremental cost against the number of contacts tested, while Figure 2B plots incremental cost against the number of persons diagnosed with HIV. Each point represents 1 simulation drawn from the parameter distributions.

For the cost per contact tested, nearly all (966/1000, 96.6%) simulations fell in the northeast quadrant, indicating that the BL implementation package was both more effective and more costly than SOC. For the cost per person diagnosed with HIV, 58% (580/1000) of simulations were in the northeast quadrant, while 42% (420/1000) were in the northwest. These findings suggest that although the BL implementation package strategy typically improves health outcomes, its cost-effectiveness is sensitive to variations in key input parameters.

### Exchange Rate Sensitivity Analysis

To assess the impact of exchange rate fluctuations during the study period, we conducted a sensitivity analysis comparing the base-case exchange rate of MWK 1036=US $1 (reflecting November 2023) with a lower exchange rate scenario of MWK 811=US $1 (reflecting November 2021). These time points correspond to the start and end of the PRACTICE study implementation period. Under the MWK 811=US $1 scenario, total program costs increased from US $111,258 to US $141,910, and cost-effectiveness estimates increased from US $125 to US $159 per contact tested and from US $2045 to US $2609 per person diagnosed with HIV. These findings indicate a moderate effect of exchange rate variability on program costs and cost-effectiveness. Multimedia Appendix 7 [36] provides additional context and assumptions.

### Scenario Analyses

Removing strategy development costs reduced the total program cost from US $111,258 in the base case to US $61,774 (Table 3). This led to a substantial decrease in the estimated ICER, with the cost per contact tested falling by US $56 (from US $125 to US $69) and the cost per person diagnosed with HIV declining by US $909 (from US $2045 to US $1136).

In the pared-down, government-led, nationwide scale-up scenario, annual program costs increased to US $727,113, while the cost per contact tested decreased to US $43 and the cost per person diagnosed with HIV declined to US $698.

**Figure 3:**
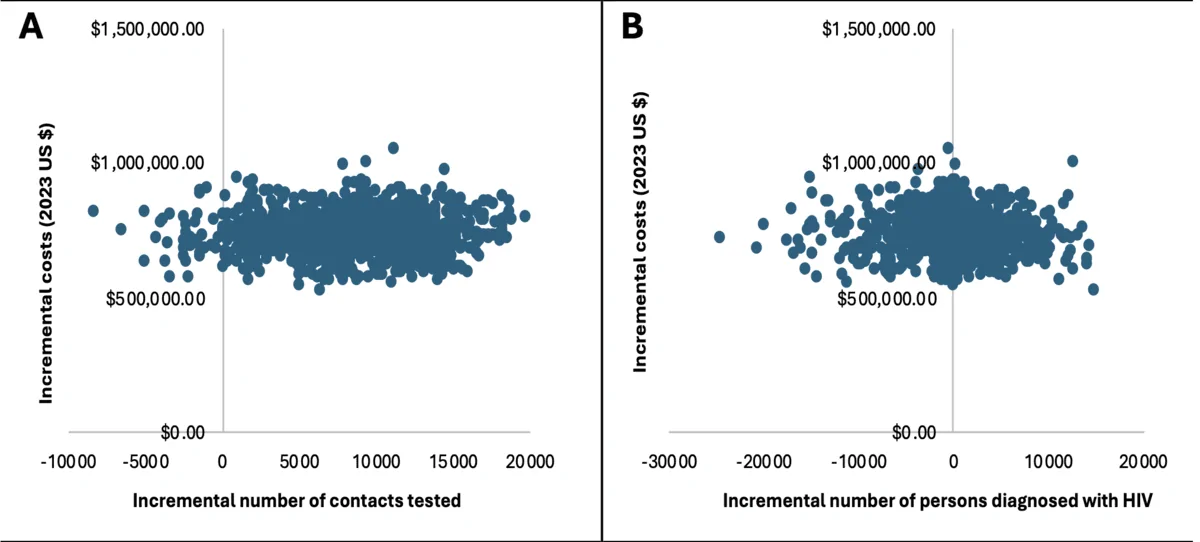
Incremental cost-effectiveness ratio (ICER) plane (nationwide expansion scenario) showing (A) incremental cost vs contact tested and (B) incremental cost vs persons diagnosed with HIV. The 4 quadrants on the ICER plane indicate different cost-effectiveness outcomes: northeast (more costly, more effective), northwest (more costly, less effective), southeast (less costly, more effective), and southwest (less costly, less effective). This figure presents the nationwide expansion scenario summarized in Table 3.

Figure 3 shows the results of the probabilistic sensitivity analysis for the nationwide expansion scenario, using the same ICER plane structure described above. For the cost per contact tested, 96.4% (964/1000) of simulations fell in the northeast quadrant, indicating that SOC + BL was more effective and more costly than SOC. For the cost per person diagnosed with HIV, just over half (552/1000, 55.2%) of simulations were in the northeast quadrant and 44.8% (448/1000) were in the northwest quadrant. No simulations fell in the southeast quadrant, indicating minimal potential for scenarios in which SOC + BL would result in both cost savings and improved outcomes relative to SOC.

## Discussion

Building on the important clinical outcomes that improved with the BL implementation package [29,30,37], our model suggests that SOC + BL is a cost-effective strategy relative to SOC for improving contact testing and HIV case finding. National expansion scenarios support additional efficiencies of scale. While similar training approaches have been assessed in other contexts [11,38,39], our findings provide unique evidence of the economic value of integrating BL into existing HCW training for HIV testing and case-finding services. The results suggest that BL training can strengthen ICT delivery at a modest cost increase compared with the current SOC.

This study is the first to examine the cost-effectiveness of a digital or blended training intervention on patient-level outcomes. BL has been implemented across various health training contexts and has generally been found to be a less expensive strategy than traditional face-to-face training [6,38], with additional cost savings observed with multiple rounds of training [39]. However, these studies focused on proxy measures of effectiveness, such as provider competency, without directly measuring patient-level benefits. We observed that cost-effectiveness was strongly influenced by participation in ICT and uptake of active referral methods, both of which were enhanced with the training provided in the BL strategy.

We included development costs to accurately portray all costs associated with this strategy [40]. Cost-effectiveness improves when these fixed, one-time investments are either spread across broader implementation contexts or excluded from ongoing programmatic analyses. Excluding one-time development costs reduced total program costs by nearly half and substantially improved cost-effectiveness, with ICERs falling from US $125 to US $69 per contact tested and from US $2045 to US $1136 per person diagnosed with HIV. Although we did not model multiple years of implementation, we observed that these effectiveness outcomes were maintained in the second year of follow-up. Therefore, we expect that the observed ICERs would decrease when subsequent years of program implementation are modeled.

Scenario analysis provided additional insight into the potential cost dynamics and scalability of SOC + BL. A pragmatic, government-led nationwide scale-up scenario demonstrated the potential for further efficiencies through integration within existing MOH structures. As expected, expanding to nearly 900 ART facilities in all 29 districts increased total program costs for 1 year of operation. However, ICERs decreased further to US $43 per contact tested and US $698 per person diagnosed with HIV. These results suggest that decentralizing training delivery and leveraging existing staff and facilities could substantially reduce per-unit costs. However, it is important to note that our trial was conducted at sites that had support from the implementing partner, Tingathe. This represents a limitation to the generalizability of our findings, as the local implementation context may change as the funding environment from external donors shifts [41-43].

Our findings are sensitive to the epidemiologic context and related parameters and can be contextualized using benchmarks from other HIV testing and partner services interventions. A 2013 provider-led partner notification study in Malawi estimated costs of US $19 per partner tested and US $36 per new diagnosis identified [44]. However, this study was conducted a decade earlier, when a much larger share of the population was living with HIV and unaware of their status. In higher-prevalence or higher-incidence settings, or settings with less mature HIV programs in which populations are expected to have greater unmet testing needs, the cost per diagnosis would likely be lower. Notably, as donor funding supporting countries with some of the world’s highest HIV burdens decreases, negative impacts on HIV services across the testing, prevention, and treatment cascades may change the cost-effectiveness of this BL implementation package.

One strength of our approach was that our effectiveness estimates were directly measured through a rigorous cluster-RCT, and this same research infrastructure facilitated robust microcosting for program elements. However, a limitation of the trial is that outcomes did not distinguish the type of elicited contact, namely sexual partners, children, or other household members, which may influence testing behaviors and outcomes. It is possible that targeting elicitation to more vulnerable contact populations could improve efficiency, particularly in Malawi’s mature HIV epidemic. Ultimately, our approach reflects how ICT data are tracked in programmatic settings and provides a useful starting point for future evaluations that may explore subgroup-specific outcomes. Another limitation is that the trial did not collect data on the proportion of named contacts who were successfully traced following notification. Our base-case assumption of a 100% tracing rate among named contacts in the active referral pathway may overestimate the effectiveness of active referral if tracing rates are lower in routine practice, as fewer contacts would subsequently be tested. However, when we varied tracing-related parameters over a plausible range (70%-100%) in both univariate and probabilistic sensitivity analyses, we observed that tracing assumptions had minimal influence on overall cost-effectiveness estimates.

Our findings may generalize to other HCW training opportunities in Malawi, given the use of nationally representative input parameters and staffing data. However, a limitation regarding external validity is that other LMICs may not have the same staffing models or costs, which could affect both program effectiveness and cost-effectiveness estimates. Regardless, this study offers a replicable cost collection matrix and evaluation framework to inform cost evaluations in other countries. Like all cost-effectiveness analyses, our outcomes and the potential generalizability of the observed ICERs depend on the primary effectiveness outcome. Another limitation affecting generalizability is that all PRACTICE trial sites were supported by PEPFAR implementing partners and may have benefited from additional infrastructure support. We observed tremendous fidelity to the BL implementation package, which was expected to influence ICT effectiveness, but may not be achieved in other cadres or settings. Adaptation and evaluation will be needed in contexts with different health system supports.

Unlike targeted HIV testing interventions designed for short-term service delivery, our BL implementation package was explicitly developed as a health system strengthening intervention to support sustained ICT delivery within routine MOH structures [40]. While alternative strategies to improve HIV testing uptake, such as secondary distribution of HIV self-testing kits among men who have sex with men in Uganda, have reported lower costs per new HIV diagnosis (eg, US $325 per new diagnosis and US $147 per additional diagnosis relative to SOC) [45], these interventions were not evaluated under national scale-up scenarios. In contrast, our intervention includes comprehensive provider training and infrastructure intended to support sustainable national scale-up, offering long-term value for health system strengthening.

The BL platform may generate additional efficiencies that were not captured in our analysis. Digital training materials and devices, such as tablets, could be reused across multiple modules or adapted for different cadres of HCWs and scaled with minimal marginal cost. Even accounting for expected tablet loss or malfunction, these strategies may offer cross-training efficiencies. While our model did not account for these downstream gains, the infrastructure established for ICT could be leveraged for other clinical areas, thereby improving the return on investment. Future evaluations could examine the cost-effectiveness of BL when integrated more broadly across health service delivery programs.

The digitally supported “spaced” training model offers pedagogical advantages with potential cost implications. Traditional week-long, centralized training requires substantial expenditures for travel, accommodation, per diem, and venue costs, as well as multiple consecutive days of staff time off-site—upward of US $500 per participant, not accounting for any gaps in clinic staffing. Spaced training also lowers the marginal cost of follow-up sessions, as digital “top-ups” can be delivered with minimal additional resources and service disruption once materials are developed. Evidence from adult learning research consistently shows that distributed practice and spaced repetition improve retention and skill consolidation relative to one-off intensive training [46-48]. While we did not model these downstream effects, noting them provides context for why blended training approaches may be economically attractive when implemented at scale, particularly in the current global health funding environment, in which less development assistance for health is available.

Ultimately, our findings support the cost-effectiveness of a decentralized, system-integrated scale-up of BL to facilitate improved ICT delivery and HIV case finding, strengthening HCW capacity for service delivery in Malawi and likely other similarly low-resource settings.

## Data Availability

The datasets generated or analyzed during this study are available from the corresponding author on reasonable request. Primary study data from the parent ICT implementation trial are not publicly available due to data sharing agreements and participant confidentiality restrictions but are available from the principal investigator on reasonable request and subject to appropriate data sharing agreements.

## Appendix

Multimedia Appendix 1 Summary of cost categories and components.

Multimedia Appendix 2 Contact tracing cost and workforce estimation methods.

Multimedia Appendix 3 Nationwide scenario: expanded methods.

Multimedia Appendix 4 Scenario analysis: partial reuse of content development and video production materials.

Multimedia Appendix 5 One-way sensitivity analyses comparing standard and blended learning index case testing implementation strategies.

Multimedia Appendix 6 One-way sensitivity analysis.

Multimedia Appendix 7 Exchange rate variation sensitivity analysis.

## Abbreviations

ART: antiretroviral therapy
BL: blended learning
CFIR: Consolidated Framework for Implementation Research
CHAM: Christian Health Association of Malawi
CQI: continuous quality improvement
HCW: health care worker
HTS: HIV testing services
ICER: incremental cost-effectiveness ratio
ICT: index case testing
LMIC: low- and middle-income country
MOH: Ministry of Health
MWK: Malawian Kwacha
PEPFAR: US President’s Emergency Plan for AIDS Relief
PRACTICE: Package of Resources for Assisted Contact Tracing: Implementation, Costs, and Effectiveness
RCT: randomized controlled trial
SOC: standard of care
TOT: training of trainers
WHO: World Health Organization
