# Supplementary material for: Cost-Effectiveness of a Blended Learning Training Approach for Health Care Workers Conducting HIV Index Case Testing in Malawi: Secondary Economic Analysis of a Cluster-Randomized Controlled Trial
Source: JMIR Med Educ. 2026 Aug 27;12:e95450. doi: 10.2196/95450 (PMC13563035; doi:10.2196/95450)
Supplement: Supplementary file 1 [file mededu_v12i1e95450_app1.docx]

**Multimedia Appendix**

Summary of Cost Categories and Components

| **Cost Category** | **Description** | **Specific Cost Components** |
| --- | --- | --- |
| Digital Learning | Costs related to content development and e-learning | Content creation, filming, technology procurement including tablets, maintenance, necessary HCW time participating in digital learning |
| In-Person Training | Costs for face-to-face training sessions | Facilitator time, site supervisors time, travel, HCW stipends, group exercises, stationery, printed materials, venue rentals, refreshments |
| Implementation & Continuous Quality Improvement (CQI) | Costs for ongoing support, remote supervision, and program implementation | Site supervisor time, HCW time, facilitator time, travel & lodging, technical support, content updates, supervision, communication costs (e.g., phone-based feedback) |
| *HCW: Healthcare Workers; FTF: Face-to-face trainings* | | |
