# Supplementary material for: Cost-Effectiveness of a Blended Learning Training Approach for Health Care Workers Conducting HIV Index Case Testing in Malawi: Secondary Economic Analysis of a Cluster-Randomized Controlled Trial
Source: JMIR Med Educ. 2026 Aug 27;12:e95450. doi: 10.2196/95450 (PMC13563035; doi:10.2196/95450)
Supplement: Supplementary file 2 [file mededu_v12i1e95450_app2.docx]

**Multimedia Appendix**

**Contact Tracing Cost and Workforce Estimation Methods**

To estimate the cost and full-time equivalent (FTE) requirements for contact tracing, we developed separate equations for phone-based and physical tracing based on distinct time and motion forms. Calculations incorporate observed time per tracing activity, healthcare worker (HCW) wage rates, and the expected annual volume of contacts. All time estimates were translated into costs using HCW salaries, and total hours were annualized to inform district-level workforce planning.

**Data Source**

- [*ACT.PhoneTracing time and motion form_29Aug2022.docx*](https://adminliveunc-my.sharepoint.com/:w:/r/personal/rutstein_ad_unc_edu/Documents/PRACTICE-R01/CEA/CEA%20Manuscript%20Development/Appendix%20Material/ACT.PhoneTracing%20time%20and%20motion%20form_29Aug2022.docx?d=wdcd2fb74b2c84b85bae3856c7b36986c&csf=1&web=1&e=zeEtGF)
- [*ACT.PhysicalTracingTime and Motion Form.29Aug2022. -*](https://adminliveunc-my.sharepoint.com/:w:/r/personal/rutstein_ad_unc_edu/Documents/PRACTICE-R01/CEA/CEA%20Manuscript%20Development/Appendix%20Material/ACT.PhysicalTracingTime%20and%20Motion%20Form.29Aug2022.%20-.docx?d=w0f05482060c74e4286173c9a18136dc8&csf=1&web=1&e=Mx2qVF)

**Equations and Definitions**

**Let:**

- *T* = Median time per contact tracing activity (in hours)
- *W* = Hourly wage of a healthcare worker
- *N* = Number of contacts traced annually
- *E* = Additional expenses per contact (e.g., transport for physical tracing)
- *H* = Total HCW time required annually
- *C*= Total cost
- *FTE*= Full-time equivalents required (assumed 2080 working hours/year)

1. **Phone-Based Tracing**

**Form:** [*ACT.PhoneTracing time and motion form_29Aug2022.docx*](https://adminliveunc-my.sharepoint.com/:w:/r/personal/rutstein_ad_unc_edu/Documents/PRACTICE-R01/CEA/CEA%20Manuscript%20Development/Appendix%20Material/ACT.PhoneTracing%20time%20and%20motion%20form_29Aug2022.docx?d=wdcd2fb74b2c84b85bae3856c7b36986c&csf=1&web=1&e=zeEtGF)

Time per contact includes: preparation, phone call, documentation, consultation, and other observed sub-activities.

*T*_phone_=*T*_prep_+*T*_call_+*T*_record_+*T*_consult_+*T*_other_

*C*_phone_=*T*_phone_×*W*×*N*_phone_

*FTE*_phone_= (T_phone_×N_phone_)/2080

2. **Physical Tracing**

**Form:** [*ACT.PhysicalTracingTime and Motion Form.29Aug2022. –*](https://adminliveunc-my.sharepoint.com/:w:/r/personal/rutstein_ad_unc_edu/Documents/PRACTICE-R01/CEA/CEA%20Manuscript%20Development/Appendix%20Material/ACT.PhysicalTracingTime%20and%20Motion%20Form.29Aug2022.%20-.docx?d=w0f05482060c74e4286173c9a18136dc8&csf=1&web=1&e=Mx2qVF)

Time includes travel time between stops and time spent tracing contacts in person. Expenses include HCW out-of-pocket costs (e.g., bike hire).

*C*_physical_= (*T*_trip_×*W*×*E*_trip_) × *N_physical_*

*FTE*_physical_= (*T*_trip_×*N*_physical_)/2080

3. **Total Tracing Resource Requirements**

*C*_total_= *C*_phone_×*C_physical_FTE*_total_ = *FTE_phone_ + FTE_physical_*

4. **Wage Calculation for Cost Inputs**

Community health worker monthly salaries ranged from 202,000 MWK to 490,000 MWK. Using an exchange rate of 1 USD = 1036 MWK, this corresponds to $195 to $472 USD per month. Assuming 173.2 working hours per month, the equivalent hourly wage ranged from $1.13 to $2.73. A midpoint value of $1.93 per hour was used for the base-case cost calculations.

5. **Tracing Cost Calculation Method**

The cost per contact for physical tracing was calculated by multiplying the median time spent tracing one person by the HCW hourly wage. Based on a sample of 747 physical tracing records, the median tracing time was 28 minutes (0.47 hours) for the Enhanced arm and 34 minutes (0.57 hours) for the Standard arm. No transport costs were included in the base-case.

Using a midpoint wage of $1.93 per hour, the estimated cost per person traced was:

- SOC + BL: $0.90
- SOC: $1.09

These unit costs were incorporated dynamically into the decision tree model by multiplying the per-contact cost by the number of individuals traced in each arm. Phone tracing costs could not be calculated at this time, as time-and-motion data were not specifically labeled for contact tracing activities.

**Workforce Time and FTE Calculation**

To estimate the total HCW effort and staffing needed for tracing activities, we multiplied the median tracing time per contact by the number of contacts traced in each arm. The total hours were divided by 2080 working hours per year to calculate FTE requirements:

Total hours= *Time per contact (T)*No. of contacts traced (N); FTE= (T*N)/2080*

In our base-case, these were the tracing outputs for our two arms:

| **Tracing Outputs** | | | |
| --- | --- | --- | --- |
| **Arm** | **Unit tracing cost ($)** | **No. of contacts traced** | **Total tracing cost ($)** |
| SOC | 1.09 | 2587.20 | 2820.05 |
| SOC + BL | 0.90 | 3968.40 | 3571.56 |

| **Total hours spent on tracing by arm** | | | |
| --- | --- | --- | --- |
| **Arm** | **Contacts Traced** | **Time per Contact (hrs)** | **Total Hours** |
| SOC | 3,968.40 | 0.47 (28 minutes) | 1,864.15 |
| SOC + BL | 2,587.20 | 0.57 (34 minutes) | 1,474.70 |
