# Supplementary material for: Cost-Effectiveness of a Blended Learning Training Approach for Health Care Workers Conducting HIV Index Case Testing in Malawi: Secondary Economic Analysis of a Cluster-Randomized Controlled Trial
Source: JMIR Med Educ. 2026 Aug 27;12:e95450. doi: 10.2196/95450 (PMC13563035; doi:10.2196/95450)
Supplement: Supplementary file 3 [file mededu_v12i1e95450_app3.docx]

**Multimedia Appendix**

**Expanded Methods – Nationwide Scenario**

The nationwide rollout was modeled using a decentralized training cascade. A four-day central TOT was included and the TOT cohort consistent of district-level leadership teams, including one District Health Officer, ART Coordinator, HTS Coordinator, and driver from each district, along with four MOH staff and national trainer. DSA and transport costs were applied for this central TOT. District trainers would then conduct facility-level trainings within their own districts, eliminating the need for per diems or overnight lodging for most sites (approximately 15% of trainers were assumed to come from outside the district and require overnight accommodation). No additional payments were included for trainers, coordinators, or site supervisors, as these activities were assumed to fall within their standard job responsibilities. Similarly, CQI activities, including individualized orientations for CQI site supervisors, CQI sessions for HCWs, and post-session debriefs, were treated as part of existing job duties, without additional compensation. For the digital training component, no additional payment or DSA was included for HCWs, as it was assumed that digital modules would be completed on site during working hours or self-directed time.

For CQI site-supervisor orientations and facility-level trainings, national estimates were derived by scaling frequency and participant numbers based on district and facility counts, while retaining core operational costs (e.g., venue and refreshments). Fuel and venue costs were substantially reduced compared to the base case, reflecting district-led implementation with limited travel and the use of MOH-owned or subsidized training spaces. Development costs were excluded, assuming use of already-developed materials for national rollout. An additional scenario incorporating reduced development costs for updating existing materials in presented in Additional file 3.

Cost inputs were scaled according to the nature of each category: one-time development costs were treated as fixed and excluded from the national model, while recurring implementation costs (e.g., travel, refreshments, and accommodation) were scaled by the number of relevant units such as training events, facilitators, or facilities. These assumptions approximated resources needs at scale while maintaining transparency in our estimation approach.

All cost data collected during the enhanced implementation phase (2023) were converted from Malawi MWK to USD using the 2023 average exchange rate of 1 USD = 1,036 MWK. For the TOT component, which was implemented and costed more recently as part of the nationwide scale-up scenario, we applied the current exchange rate of 1 USD = 1,751.12 [50]. This approach ensured temporal consistency between cost data and the exchange rate applicable at the time of expenditure.
