# Supplementary material for: Cost-Effectiveness of a Blended Learning Training Approach for Health Care Workers Conducting HIV Index Case Testing in Malawi: Secondary Economic Analysis of a Cluster-Randomized Controlled Trial
Source: JMIR Med Educ. 2026 Aug 27;12:e95450. doi: 10.2196/95450 (PMC13563035; doi:10.2196/95450)
Supplement: Supplementary file 4 [file mededu_v12i1e95450_app4.docx]

**Multimedia Appendix**

**Scenario Analysis: Partial reuse of content development and video production materials**

We examined an additional scenario in which content development and video production costs were reduced by 50% to reflect anticipated savings from reusing or updating existing materials in future implementations. This adjustment simulates a setting where foundational assets—such as scripts, animations, and core video content—would not require full redevelopment. Under this scenario, the total program cost decreased from $111,258 to $94,899. These reductions yielded more modest improvements in cost-effectiveness, with the ICER decreasing from $125 to $107 per contact tested and from $2,045 to $1,745 per PWH diagnosed.
