# Supplementary material for: Cost-Effectiveness of a Blended Learning Training Approach for Health Care Workers Conducting HIV Index Case Testing in Malawi: Secondary Economic Analysis of a Cluster-Randomized Controlled Trial
Source: JMIR Med Educ. 2026 Aug 27;12:e95450. doi: 10.2196/95450 (PMC13563035; doi:10.2196/95450)
Supplement: Supplementary file 5 [file mededu_v12i1e95450_app5.docx]

**Multimedia Appendix**

One-way sensitivity analyses comparing standard and blended learning ICT Implementation Strategies

| **Parameter** |  | | **Input** | | **$/contact tested** | | **$/PWH diagnosed** | |
| --- | --- | --- | --- | --- | --- | --- | --- | --- |
|  | **Base-case** | **Lower bound** | | **Upper bound** | **Low ICER** | **High ICER** | **Low ICER** | **High ICER** |
| Probability of index identification via HTS (SOC and SOC+BL) | 6.60% | 6.60% | | 18.20% | 124.84 | 109.09 | 2045.12 | 1810.55 |
| Index identified via HTS and participating in ICT (SOC + BL) | 97.11% | 77.60% | | 100.00% | 167.77 | 120.30 | 3320.01 | 1935.43 |
| Index identified via ART and participating in ICT (SOC + BL) | 73.09% | 58.40% | | 87.60% | 217.00 | 88.17 | 2888.17 | 1590.57 |
| Index identified via HTS and participating in ICT (SOC) | 92.93% | 74.40% | | 100.00% | 101.99 | 136.56 | 1512.41 | 2364.01 |
| Index identified via ART and participating in ICT (SOC) | 44.70% | 36.00% | | 54.00% | 100.38 | 169.10 | 1835.29 | 2331.61 |
| Contacts elicited via Active referral (SOC+BL / HTS) | 33.40% | 26.40% | | 39.60% | 172.83 | 126.91 | 5985.02 | 2645.37 |
| Contacts elicited via passive referral with self-test kits (SOC+BL / HTS) | 7.46% | 5.60% | | 8.40% | 150.85 | 142.19 | 3964.85 | 3417.73 |
| Contacts elicited via passive referral without self-test kits (SOC+BL / HTS) | 59.14% | 47.20% | | 70.80% | 158.47 | 133.86 | 4537.78 | 2972.77 |
| Contacts elicited via Active referral (SOC+BL / ART) | 47.69% | 38.40% | | 57.60% | 171.73 | 96.86 | 2516.3 | 1706.99 |
| Contacts elicited via passive referral with self-test kits (SOC+BL / ART) | 13.19% | 10.50% | | 15.80% | 134.48 | 116.70 | 2151.57 | 1951.18 |
| Contacts elicited via passive referral without self-test kits (SOC+BL / ART) | 39.13% | 31.20% | | 46.80% | 134.52 | 116.68 | 2152.08 | 1950.98 |
| Contacts elicited via Active referral (SOC/HTS) | 32.82% | 31.00% | | 100.00% | 120.58 | -376.28 | 1937.56 | -1859.57 |
| Contacts elicited via passive referral with self-test kits (SOC/HTS) | 7.35% | 5.60% | | 8.40% | 121.07 | 127.18 | 1950.24 | 2106.13 |
| Contacts elicited via passive referral without self-test kits (SOC/HTS) | 59.83% | 30.00% | | 100.00% | 105.78 | 164.74 | 1595.24 | 3295.84 |
| Contacts elicited via Active referral (SOC/ART) | 37.73% | 30.00% | | 46.00% | 108.68 | 148.58 | 1912.68 | 2209.27 |
| Contacts elicited via passive referral with self-test kits (SOC/ART) | 13.22% | 10.00% | | 16.00% | 118.12 | 131.25 | 1991.21 | 2093.56 |
| Contacts elicited via passive referral without self-test kits (SOC/ART) | 49.05% | 39.00% | | 59.00% | 117.71 | 132.75 | 1987.88 | 2104.6 |
| Individuals tested via Active referral | 49.00% | 40.00% | | 62.00% | 145.22 | 103.76 | 2353.38 | 1719.3 |
| HIV Positivity (SOC+BL / HTS) | 9.14% | 9.14% | | 73.70% | 124.84 | 124.84 | 2045.12 | 140.53 |
| HIV Positivity (SOC/HTS) | 9.56% | 9.56% | | 73.70% | 124.84 | 124.84 | 2045.12 | -183.67 |
| Individuals tested via passive referral with self-test kits | 44.00% | 16.00% | | 71.00% | 136.68 | 115.18 | 2259.64 | 1873.19 |
| Individuals tested via passive referral without self-test kits | 15.00% | 15.00% | | 34.00% | 124.84 | 110.93 | 2045.12 | 1742.66 |
| HIV Positivity (SOC+BL / ART) | 4.21% | 4.21% | | 16.00% | 124.84 | 124.84 | 2045.12 | 400.78 |
| HIV Positivity (SOC/ART) | 2.90% | 2.90% | | 16.00% | 124.84 | 124.84 | 2045.12 | -1187.8 |
| Probability of contacts being traced across all groups | 100.00% | 70.00% | | 100.00% | 124.84 | 124.84 | 2045.12 | 2045.12 |
| Cost of content development (strategy development) | 22000.00 | 17600.00 | | 26400.00 | 119.89 | 129.75 | 1964.05 | 2125.68 |
| Cost of video production (strategy development) | 10718.51 | 8574.81 | | 12862.21 | 122.42 | 127.22 | 2005.49 | 2084.24 |
| Cost of capital purchases (strategy development)) | 14951.19 | 11213.39 | | 18688.99 | 120.63 | 129.01 | 1976.22 | 2113.52 |
| Cost of office supplies (strategy development) | 1428.33 | 1142.66 | | 1714.00 | 124.50 | 125.14 | 2039.62 | 2050.12 |
| Cost of miscellaneous items (strategy development) | 386.10 | 308.88 | | 463.32 | 124.73 | 124.91 | 2043.45 | 2046.29 |
| Cost of FTF site-supervisor orientation (implementation) | 3875.77 | 2713.04 | | 5038.50 | 123.49 | 126.15 | 2023.09 | 2066.65 |
| Cost of individual tablet-guided learning sessions (implementation) | 7839.07 | 6271.26 | | 9406.88 | 123.06 | 126.58 | 2016.07 | 2073.66 |
| Cost of small-group face-to-face practice sessions (implementation) | 31950.07 | 22365.05 | | 41535.09 | 114.07 | 135.56 | 1868.82 | 2220.92 |
| Cost of one-on-one phone-based feedback (implementation) | 2013.37 | 1510.03 | | 2516.71 | 124.25 | 125.38 | 2035.62 | 2054.11 |
| Cost of continuous quality improvement orientation (face-to-face and remote) (implementation) | 3393.70 | 2714.96 | | 4072.44 | 124.06 | 125.58 | 2032.4 | 2057.33 |
| Cost of continuous quality improvement sessions and remote debrief (implementation) | 11927.10 | 7752.62 | | 16101.59 | 120.14 | 129.50 | 1968.2 | 2121.54 |
| Cost of tracing per contact (SOC) | 1.09 | 0.87 | | 1.31 | 125.44 | 124.20 | 2055.07 | 2034.67 |
| Cost of tracing per contact (SOC + BL) | 0.90 | 0.72 | | 1.08 | 124.03 | 125.61 | 2031.92 | 2057.82 |
