# Supplementary material for: Cost-Effectiveness of a Blended Learning Training Approach for Health Care Workers Conducting HIV Index Case Testing in Malawi: Secondary Economic Analysis of a Cluster-Randomized Controlled Trial
Source: JMIR Med Educ. 2026 Aug 27;12:e95450. doi: 10.2196/95450 (PMC13563035; doi:10.2196/95450)
Supplement: Supplementary file 6 [file mededu_v12i1e95450_app6.docx]

**Multimedia Appendix**

One-way sensitivity analyses of key input parameters.

(a) One-Way Sensitivity Analysis on Cost per Contact Tested

Change in ICER

|  | Min Change | Max Change |
| --- | --- | --- |
| Index identified via ART and enrolled in ICT* | -34.36 | 34.36 |
| Contact elicited by passive referral without self-test kits (HTS)* | -29.48 | 29.48 |
| Contacts elicited via Active referral (ART)* | -19.95 | 19.95 |
| Index identified via HTS and enrolled in ICT* | -17.285 | 17.285 |
| Cost: Face-to-face practice sessions | -10.745 | 10.745 |
| Contacts elicited via passive referral without self-test kits (ART)* | -7.52 | 7.52 |
| Contacts elicited via passive referral with self-test kits (ART)* | -6.565 | 6.565 |
| Cost: Content development | -4.93 | 4.93 |
| Cost: Continuous Quality Improvement | -4.68 | 4.68 |
| Cost: Capital purchases | -4.19 | 4.19 |
| Contacts elicited via passive referral with self-test kits (HTS)* | -3.055 | 3.055 |

(b) One-Way Sensitivity Analysis on Cost per PWH Diagnosed

|  | Min Change | Max Change |
| --- | --- | --- |
| Contacts elicited via passive referral without self-test kits (HTS)* | -850.3 | 850.3 |
| Index identified via HTS and enrolled in ICT* | -425.8 | 425.8 |
| Index identified via ART and enrolled in ICT* | -248.16 | 248.16 |
| Cost: Face-to-face practice sessions | -176.05 | 176.05 |
| Contacts elicited via Active referral (ART)* | -148.295 | 148.295 |
| Cost: Content development | -80.815 | 80.815 |
| Contacts elicited via passive referral with self-test kits (HTS)* | -77.945 | 77.945 |
| Cost: Continuous quality improvement | -76.67 | 76.67 |
| Cost: Capital purchases | -68.65 | 68.65 |
| Contacts elicited via passive referral without self-test kits (ART)* | -58.36 | 58.36 |
| Contacts elicited via passive referral with self-test kits (ART)* | -51.175 | 51.175 |
