# Supplementary material for: Cost-Effectiveness of a Blended Learning Training Approach for Health Care Workers Conducting HIV Index Case Testing in Malawi: Secondary Economic Analysis of a Cluster-Randomized Controlled Trial
Source: JMIR Med Educ. 2026 Aug 27;12:e95450. doi: 10.2196/95450 (PMC13563035; doi:10.2196/95450)
Supplement: Supplementary file 7 [file mededu_v12i1e95450_app7.docx]

**Multimedia Appendix**

Exchange rate variation sensitivity analysis

To account for exchange rate fluctuations during the study period (November 2021 to November 2023), we conducted a sensitivity analysis on the USD–Malawi Kwacha (MWK) exchange rate. This period saw multiple devaluations of the MWK, including a drop from approximately 800 MWK/USD in late 2021 to about 1,036 MWK/USD by 2023 (ExchangeRate.com). While most implementation costs—such as personnel, transportation, and training—were incurred in MWK, content development was contracted in USD. As the implementing partner was reimbursed in USD, all costs were ultimately converted to USD at the time of expenditure. To test the impact of exchange rate assumptions on our findings, we compared our base case scenario (using 1,036 MWK/USD) with a sensitivity scenario assuming a fixed rate of 811 MWK/USD. The total program cost increased from $111,258 to $141,910 under the lower exchange rate, and the cost-effectiveness ratios rose from $125 to $159 per contact tested and from $2,045 to $2,608 per PWH diagnosed. These findings suggest that exchange rate shifts had a moderate impact on overall program costs and cost-effectiveness outcomes.
